# Deep-learning and MR images to target hypoxic habitats with evofosfamide in preclinical models of sarcoma

**DOI:** 10.7150/thno.56595

**Published:** 2021-03-11

**Authors:** Bruna V. Jardim-Perassi, Wei Mu, Suning Huang, Michal R. Tomaszewski, Jan Poleszczuk, Mahmoud A. Abdalah, Mikalai M. Budzevich, William Dominguez-Viqueira, Damon R. Reed, Marilyn M. Bui, Joseph O. Johnson, Gary V. Martinez, Robert J. Gillies

**Affiliations:** 1 Department of Cancer Physiology, Moffitt Cancer Center, Tampa, US.; 2 Current Address: Guangxi Medical University Cancer Hospital, Nanning Guangxi, China.; 3 Department of Integrated Mathematical Oncology, Moffitt Cancer Center, Tampa, US.; 4 Current Address: Nalecz Institute of Biocybernetics and Biomedical Engineering, Polish Academy of Sciences, Poland.; 5 Quantitative Imaging Core, Moffitt Cancer Center, Tampa, Florida.; 6 Small Animal Imaging Laboratory, Moffitt Cancer Center, Tampa, Florida.; 7 Department of Interdisciplinary Cancer Management, Adolescent and Young Adult Program, Moffitt Cancer Center, Tampa, Florida.; 8 Department of Pathology, Moffitt Cancer Center, Tampa, Florida.; 9 Analytic Microscopy Core, Moffitt Cancer Center, Tampa, Florida.; 10 Current Address: Department of Imaging Physics, The University of Texas M.D. Anderson Cancer Center.

**Keywords:** hypoxia, tumor microenvironment, deep learning, hypoxia-activated prodrugs, *in-vivo* imaging

## Abstract

**Rationale:** Hypoxic regions (habitats) within tumors are heterogeneously distributed and can be widely variant. Hypoxic habitats are generally pan-therapy resistant. For this reason, hypoxia-activated prodrugs (HAPs) have been developed to target these resistant volumes. The HAP evofosfamide (TH-302) has shown promise in preclinical and early clinical trials of sarcoma. However, in a phase III clinical trial of non-resectable soft tissue sarcomas, TH-302 did not improve survival in combination with doxorubicin (Dox), possibly due to a lack of patient stratification based on hypoxic status. Therefore, we used magnetic resonance imaging (MRI) to identify hypoxic habitats and non-invasively follow therapies response in sarcoma mouse models.

**Methods:** We developed deep-learning (DL) models to identify hypoxia, using multiparametric MRI and co-registered histology, and monitored response to TH-302 in a patient-derived xenograft (PDX) of rhabdomyosarcoma and a syngeneic model of fibrosarcoma (radiation-induced fibrosarcoma, RIF-1).

**Results:** A DL convolutional neural network showed strong correlations (>0.76) between the true hypoxia fraction in histology and the predicted hypoxia fraction in multiparametric MRI. TH-302 monotherapy or in combination with Dox delayed tumor growth and increased survival in the hypoxic PDX model (p<0.05), but not in the RIF-1 model, which had a lower volume of hypoxic habitats. Control studies showed that RIF-1 resistance was due to hypoxia and not other causes. Notably, PDX tumors developed resistance to TH-302 under prolonged treatment that was not due to a reduction in hypoxic volumes.

**Conclusion:** Artificial intelligence analysis of pre-therapy MR images can predict hypoxia and subsequent response to HAPs. This approach can be used to monitor therapy response and adapt schedules to forestall the emergence of resistance.

## Introduction

Sarcomas constitute an heterogeneous group of malignant tumors of mesenchymal origin, divided into two categories: soft tissue sarcomas (STS) and sarcomas of bone 1. Although STS account for only 1.5% of all malignant tumors in adults, with an estimated 13,130 new cases in the United States in 2020, they represent approximately 7.4% of all tumors in children and young adults 2, 3.

The heterogeneity of sarcomas is significant with at least 50 different histologic subtypes, all of which have distinct biologic behavior and therapy response 4. Rhabdomyosarcoma is the most common STS histological type in children, accounting for 3 to 5% of all pediatric tumors every year in the United States and 50% of all STS diagnosed in children under age 10 5. While it is typically sensitive to chemotherapy initially, durable control of the primary tumor requires surgical resection and/or radiation therapy 5, 6. Fibrosarcoma is rarer, currently accounting for 3.6% of STS 7. Regardless the subtype, however, the standard of care for non-rhabdomyosarcoma STS patients is fairly homogeneous: first-line chemotherapeutic agents, such as doxorubicin (Dox), surgery, and radiation. However, the clinical response to these drugs is heterogeneous and limited 8.

Sarcoma often presents with significant tumor hypoxia, which is associated with poor prognosis and innate biochemical resistance to chemo- and radiotherapies 9. Hypoxic tissue also associated with poor vascular perfusion, which can lead to inefficient drug delivery and hence physiological resistance 10. Hence, regional hypoxia can subsequently lead to the formation of localized environmental niches where drug-resistant cell populations can survive, evolve, and thrive. Thus, targeting hypoxia in the tumor microenvironment is of great interest to improve clinical outcome 11.

For this purpose, hypoxia-activated prodrugs (HAPs) have been designed to penetrate hypoxic regions and release cytotoxic agents. Multiple HAPs are in development with low toxicity and proven efficacy in pre-clinical and early stage clinical trials 12. Evofosfamide (TH-302) is a HAP created by linking a 2-nitroimidazole moiety to the DNA cross-linker bromo-ifosfamide mustard (Br-IPM). As with most HAPs, TH-302 is selectively reduced under hypoxic conditions, which releases the Br-IPM leading to DNA crosslinking 13. HAPs have been tested in clinical trials but despite early promise in phase I-II trials, definitive phase III trials have failed to show a survival benefit 14. Specifically, the randomized two-arm phase III clinical trial TH CR-406/SARC021 (NCT01440088) tested TH-302 + Dox *vs.* Dox in patients with locally advanced, unresectable, or metastatic soft-tissue sarcomas. Although the proportion of patients who achieved complete or partial response was significantly higher and progression free survival (PFS) was prolonged for the TH-302 + Dox arm, this combination did not improve overall survival (OS) when compared with Dox monotherapy, which was the primary endpoint 15. Some limitations have been discussed regarding the lack of OS improvement in this trial 16, 17, but one logical possibility was a complete lack of patient stratification based on hypoxic status to identify patients who were most likely to benefit from HAP therapy and simultaneously least likely to benefit from Dox monotherapy 18.

In this context, we propose that imaging of hypoxia may help with patient stratification and therapy monitoring 19-22. Pimonidazole (PIMO) is often used as the “gold standard” to measure hypoxia in tissues, but a major limitation is that it requires collection of tissue for histology, which is not conducive for longitudinal measurements. Although a biopsy can be taken to assess hypoxic status prior to or during therapy, it would not account for intratumor heterogeneity and would be prone to interfering with the study. In this context, if noninvasive imaging of hypoxia can be developed it would allow longitudinal monitoring of therapy response without a biopsy.

To this end, different magnetic resonance imaging (MRI)- and positron emission tomography (PET)-imaging based analyses have been explored to identify tumor hypoxia 23, 24 and predict response to HAPs in pre-clinical models 19, 25. We have previously reported that multiparametric (mp) data using T_2_, T_2_*, diffusion-weighted imaging (DWI) and dynamic contrast enhanced (DCE) MRI maps can capture subtle differences in the tumor microenvironments, and is able to differentiate viable, necrotic and hypoxic tumor habitats in breast cancer models 26. Herein, we take a similar approach to identify hypoxia in sarcoma using deep learning (DL) models developed with mp data from T_2_-weighted (T_2_W), T_2_ map, T_2_* map and DCE-MRI co-registered with PIMO stained histology.

The main goal of this study was to classify hypoxic habitats, in order to monitor TH-302 therapy response in preclinical models of sarcoma. Thus, this study had two specific goals: first, to evaluate the response to TH-302 monotherapy or in combination with Dox in sarcoma mouse models; and second to develop a combined DL and MRI-based method to identify hypoxic habitats to investigate the temporal evolution of changes in hypoxic habitats noninvasively in sarcomas over the course of treatment.

## Materials and Methods

### Sarcoma mouse models

Animal experiments were approved by the Institutional Animal Care and Use Committee (IACUC), and Institutional Review Board (IRB) (University of South Florida) (Protocol #4778). All mice were obtained from Charles River Laboratory (Wilmington, MA) and housed in a facility under pathogen-free conditions in accordance with IACUC standards of care at the H. Lee Moffitt Cancer Center.

Two sarcoma models were used in this study: 1) a patient-derived xenograft (PDX) of rhabdomyosarcoma and 2) a murine fibrosarcoma syngeneic model.

To develop the PDX model, cryopreserved PDX rhabdomyosarcoma cells (reference number: SJRHB010468_X1) were obtained from The Childhood Solid Tumor Network (CSTN) at St. Jude Hospital 27. Tumor was established through subcutaneous implantation into the flank of severe combined immunodeficiency (SCID) Hairless Outbread (SHO) mice (female, 6-8 weeks of age). Tumors were measured with digital caliper and were passaged into new mice when them reached >1000 mm^3^. Mice were anesthetized with 2% isoflurane delivered in 1.5 L/min oxygen ventilation and tumors were collected, placed in Roswell Park Memorial Institute (RPMI) 1640 culture media (Gibco, Waltham, MA) and dissected to 1 mm^3^ pieces. Tumors explants were then implanted into new mice with 50% RPMI1640 / 50% Matrigel.

The fibrosarcoma model was developed by inoculating the radiation-induced fibrosarcoma cell line (RIF-1) 28 into immunocompetent C3H mice (female, 6-8 weeks of age). An additional experiment was performed, where RIF-1 cells were inoculated into immunodeficient NSG (NOD scid gamma). RIF-1 cells were kindly provided by Dr. Zaver M. Bhujwalla, Department of Radiology, Johns Hopkins School of Medicine. RIF-1 cells were maintained in Waymouth's media (Gibco, Waltham, MA) supplemented with 10% fetal bovine serum (FBS), 1 mM of N-2-hydroxyethylpiperazine-N-ethanesulfonic acid (HEPES) and 1% of penicillin/streptomycin (P/S) (Sigma, St. Louis, MO) at 37 °C and 5% CO_2_. RIF-1 cells were confirmed to be of mouse origin and no mammalian interspecies contamination was detected for the sample using short tandem repeat (STR) DNA profiling. Cells were tested free of mycoplasma (MycoAlert Mycoplasma Detection kit; Lonza, Basel, Switzerland). For tumor inoculation, RIF-1 cells were suspended in Hanks' Balanced Salt Solution (HBSS) media (Gibco, Waltham, MA) and 1 × 10^6^ cells were subcutaneously inoculated in the right flank of mice.

### Groups of treatment

Tumor volumes were measured by acquiring multi-slice axial T_2_W MRI covering the entire tumor (TurboRARE sequence; repetition time (TR) = 4825 ms, effective echo time (TE) = 73.58 ms, field of view (FOV) = 35 × 35 mm^2^, matrix = 256 × 256, slice thickness of 1 mm). Tumor volumes were obtained from manually drawn regions of interest (ROIs) in MATLAB (MathWorks, Natick, MA) using an open source toolbox for medical image analysis (aedes.uef.fi). During MRI scanning, mice were maintained anesthetized with 2% isoflurane delivered in 1.5 L/min oxygen ventilation, and body temperature and respiratory function were continuously monitored (SA Instruments Inc, System 1025, Stony Brook, NY) and maintained at 37 °C ± 0.7 °C and 40-60 breaths per min, respectively.

When tumors reach approximately 500 mm^3^ (day 0), mice were randomly assigned to the following treatment groups: 1) Control; 2) monotherapy with Dox, dose of 4 mg/kg by intravenous (IV) injection once a week; 3) monotherapy with TH-302 (obtained from Threshold Pharmaceuticals, Redwood City, CA), dose of 50 mg/kg by intraperitoneal (IP) injection, five times per week; and 4) combination of TH-302 + Dox.

The percentage of tumor growth changes and OS were calculated from the first day of treatment (day 0) until the last of experiment, when individual tumors reached approximately 1500 mm^3^.

For the PDX model, a total of 22 SCID/SHO mice were studied with 3 mice dying during the experiment in the MRI scanner, and were excluded from the study. Treatment groups were composed of 5; 5; 5 and 4 mice in Control; Dox; TH-302; and TH-302 + Dox groups, respectively. For the RIF-1 model, 20 C3H mice were inoculated with tumor cells, while one mouse did not develop a tumor and one died during MRI scanning and was excluded from the study. Treatment groups were composed of 4; 5; 5; and 4 mice in Control; Dox; TH-302; and TH-302 + Dox groups, respectively. For the additional experiment with immunodeficient NSG mice, 5 mice were used in total, where RIF-1 cells were inoculated into those mice, 2 were used as control and 3 treated with TH-302 monotherapy.

### Cell viability *in vitro*

Cell viability was assessed by crystal violet (Sigma, St. Louis, MO) to test the *in vitro* response to different concentration of TH-302 in hypoxic conditions and to a DNA cross-linking agent mitomycin C (MCC) (Tocris, Minneapolis, MN). Experiments were performed with RIF-1 cells and rhabdomyosarcoma PDX-derived dissociated cells, as well as with cell lines of human rhabdomyosarcoma (RD) (ATCC, Manassas, VA) and human lung cancer (H460) (ATCC, Manassas, VA), which were used as positive controls.

To obtain the dissociated PDX cells, tumors were harvested and enzymatically disassociated using the Animal free Collagenase/Dispase Blend II reagent (Millipore, Burlington, MA). Cells were maintained in culture with RPMI-1640 media (Gibco, Waltham, MA) supplemented with 10% FBS and 1% of P/S at 37 °C and 5% CO_2_.

Cells were seeded into 96-well plates and grown overnight prior to initiating treatment. For TH-302 experiments, on the day of the test, cells were exposed to increasing concentrations of TH-302 and the plates were incubated overnight under normoxic (20% O_2_) or hypoxic conditions (0.2% O_2_ and 1% O_2_). After overnight exposure, plates were removed from the hypoxia chamber and further incubated for 72 h in standard incubator (20% O_2_). For MCC experiments, cells were treated with different concentrations of MCC for 72 h under normoxia (20% O_2_).

Cells were washed with PBS, fixed with 100% methanol for 10 min, and stained with 0.5% crystal violet solution in 25% methanol for 10 min. The crystal violet solution was discarded, and cells were washed with water and allow to dry at room temperature (RT). The stain was solubilized with 1% sodium dodecyl sulfate (SDS) and plate was placed in an orbital shaker until color was uniformly distributed in each well. Absorbance (abs) was read at 540 nm. Cell viability (%) was calculated using the formula (%) = [100*(sample abs)/ (control abs)].

### Multiparametric MRI (mpMRI)

mpMR images (T_2_W, T_2_ map, T_2_* map and DCE-MRI) were acquired pre- and post- therapy. Imaging was acquired for each mouse at day 0 (pre-therapy) and longitudinally until the last day of therapy.

Imaging acquisition and corresponding MR parametric maps were obtained similarly as described in 26. T_2_ and T_2_* maps were generated with the multi slice multiecho (MSME) and multi gradient echo (MGE) sequences, respectively. T_1_-weighted DCE-MRI were acquired pre- and post- IV administration of 0.2 mmol/kg gadobutrol (Gadavist; Bayer, Leverkusen, Germany). All sequences were obtained with FOV of 35 × 35 mm^2^, matrix size of 256 × 256, 11 central slices with a slice thickness of 1 mm. Imaging was performed using a 35 mm Litzcage coil (Doty Scientific, Inc, Columbia, SC) on a 7T horizontal magnet (Agilent ASR 310, Santa Clara, CA) and NMR platform (Bruker Biospin, Inc. BioSpec AV3HD, Billerica, MA). T_2_ and T_2_* maps were computed in ParaVision 6.0.1 (Bruker Biospin, Inc, Billerica, MA). The total acquisition time for DCE-MR imaging was 25 min and 66 s, and the temporal resolution was approximately 70 s per scan, with 22 repetitions. Gadobutrol was administered through an IV catheter after the first repetition. Semi-quantitative parametric maps calculated from DCE-MRI data included area under the time-series curve (AUC), slope and time to maximum (TTM). The AUC is the sum of the entire DCE dynamic curve, not only the initial uptake as commonly report with iAUC, allowing extra time for some regions to shown slow uptake, which could be indicative of hypoxia 26. The slope is the numerical ratio: ΔS_T1_/Δt, where ΔS is the T_1_-weighted signal intensity temporal change and Δt is the corresponding time-difference and the TTM is the time that corresponds to the maximum enhancement achieved.

### Histology

To ensure the co-registration of histology with MRI, tumors were collected after the last mpMRI, according with the 3D-printed tumor mold workflow developed previously 26. Briefly, multi-slice axial T_2_-weighted images were acquired during the last mpMRI scanning (a slice thickness of 1 mm, FOV of 35 × 35 mm^2^ and image size of 256 × 256), and a ROI was drawn encompassing the entire tumor to create a 3D-printed tumor mold. Tumor-specific molds were designed in SolidWorks (Dassault Systems, SolidWorks Corp., Waltham, MA), containing slots every 2 mm, which were used to guide the slicing of the tumor, aligned with the 1 mm MRI slices. Thus, each tumor was sliced in serial 2 mm tissues, placed in individual cassettes, and embedded in paraffin to perform immunohistochemistry (IHC) staining.

Paraffin tissue-blocks were serially sectioned with slices thickness of 4 µm on a microtome (Leica Biosystem, Buffalo Grove, IL) and allowed to dry at RT and subsequently heated to 60 °C for 1 h.

PIMO was used as a hypoxia marker, as it is an exogenous 2-nitroimidazole probe that binds covalently to thiol-containing proteins when the O_2_ tension is below 10 mmHg (< 1.3%) 29 and can be visualized in histological sections by IHC. Mice received an IP injection of PIMO hydrochloride (60 mg/kg) 1 h prior to collection of tumors, and PIMO staining was detected by IHC using an anti-PIMO antibody (PAB2627AP, HPI, Burlington, MA). In addition, IHC was performed with the following primary antibodies: Cluster of differentiation 31 (CD31) (#ab28364, Abcam, Cambridge, MA), which was used as a marker of endothelial cells of blood vessels; Cleaved Caspase-3 (CC3) (#9661, Cell Signaling, Danvers, MA) used as an apoptosis marker; and phospho gamma-H2AX (#NB100-2280, Novus Biologicals, Littleton, CO) used as a marker for DNA-damage.

IHC protocol consisted in deparaffinization of slides in xylene and hydration through subsequently incubation in aqueous solutions of decreasing ethanol concentration. Endogenous peroxidase activity was blocked with 0.6% H_2_O_2_ in methanol for 30 min and antigen retrieval with citrate buffer (pH 6.0) in the pressure cooker for 20 min. Sections were incubated with 10% goat serum at 4 °C overnight for blocking, followed by incubation with primary antibody in humid chamber for 1 h at RT, and with a biotinylated secondary antibody (Vectastain Elite kit; Vector Labs, Burlingame, CA) for 60 min at RT. Section were then incubated in avidin-biotin complex (ABC) (Vectastain Elite ABC Kit; Vector Labs, Burlingame, CA) for 45 min and chromogen substrate visualization was performed using the NovaRed VectaStain Peroxidase kit (Vector Labs SK-4800, Burlingame, CA).

Sections were counterstained with hematoxylin, dehydrated in ethanol followed by xylene, and finally mounted using Permount medium (Thermo Scientific, Waltham, MA). Negative controls were obtained by omitting the primary antibody, and a tissue known to express the protein of interest was used as positive controls in every assay. Positive controls for each antibody were: MDA-MB-231 breast cancer for PIMO for both human (PDX) and mouse (RIF-1) tissues; placenta and tonsil for CD31 antibody for mouse and human tissues, respectively; spleen and tonsil for CC3 antibody for mouse and human tissues, respectively; and colon adenocarcinoma for phospho gamma-H2AX, for both mouse and human tissues.

### Histological analyses

#### Detection and quantification of positive pixels

Histology slides were scanned (Aperio AT2, Leica Biosystems, Buffalo Grove, IL), saved as .svs files and imported into Visiopharm software (Visiopharm A/S, Horsholm, Denmark).

Multiple intensity-based threshold algorithms were created to identify positive stained-pixels for each antibody. A global threshold was used across all images for each antibody (CD31, CC3 and phospho gamma-H2AX). Area of positive pixels (%) (stained pixels) was calculated over the total area. For CD31, microvessel density was quantified by calculating the number of vessels by unit area (mm^2^). The results were verified by the study pathologist.

### Binary mask of pimonidazole positive areas

For PIMO staining, MATLAB was used to automatically select individual thresholds based on the Otsu method for each histological slice. Threshold levels were calculated in grayscale images, on a scale from 0 (no staining, white) to 255 (maximum staining, black) (**Figures S1A-B**).

Then, Visiopharm software was used to create the binary mask of PIMO-positive areas using individual Otsu threshold-based algorithms for each slice. The fractional PIMO-positive area was calculated for each slice. To compare PIMO-positive area in histology between groups of therapy, values of all histological slices for each tumor were averaged to have one value per tumor. To ensure that the individual thresholding method would not affect the comparison of PIMO-positive areas between groups of therapy, we calculated a global threshold across all PIMO-stained slices for each tumor type. Using these global thresholds (92 for PDX and 86 for RIF-1), we recalculated the PIMO-positive area for each tumor (**Figure S1C**). Notably, this alternative thresholding method did not significantly affect the values of total PIMO-positive area in each tumor, nor the comparison between groups (**Figure S1D**). Individual thresholds were chosen to increase accuracy in detecting PIMO positive areas as it was used as the true hypoxia fraction for the convolutional neural network (CNN) models.

### MRI and histology co-registration algorithm

MATLAB was used to convert the full resolution binary masks of PIMO-positive pixels generated in VisioPharm software (.mld files) into .mat files, which was then co-registered with MRI slices and used as input to the build DL models to identify hypoxic habitats.

Information about positive pixel areas in .mld files is stored as a collection of polygons. Each polygon is defined by a list of consecutive vertices, which are connected by lines. The order of the polygons defines which one encompasses a positive region and which one is a boundary of a negative one (e.g. hole). In order to transform that information into an image of given resolution and store it as a .mat file in MATLAB, each polygon was drawn into an image matrix using Bresenham's line algorithm and then filled accordingly using queue-scanline algorithm going from top of the image to bottom.

The PIMO-positive mask for each histology slice was co-registered with the mp-maps of the corresponding MRI slice, according to a method previously described in 30. Briefly, custom written MATLAB code was used to perform affine 2D registration based on manual detection of 4 corresponding landmarks in histology and MRI images. Prior to co-registration, slices where the tissues were broken or with missing parts were excluded from co-registration analyses.

Dice similarity coefficients (DSC) were calculated between each MRI slice and its corresponding histology slice by creating binary masks for both slices and measuring the similarity between the masks using Dice formula in equation 1. The similarity score ranges between 0 and 1. A score close or equal to 1 indicates the slices are very similar or identical. More specifically, if ***M*** is the binary mask of the MRI slice and ***H*** is the histology binary mask, the DSC score is obtained by the following Dice equation:


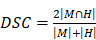
(1)

### Hypoxic habitats using deep-learning model

As described above, the ground truth hypoxia maps were the mask generated from PIMO stained histology. These were then down sampled and co-registered with the corresponding mpMRI maps of T_2_W, T_2_ map, T_2_* map, slope, TTM and AUC images, and a DL model was used to identify which combination of the MR parameters best predicted hypoxia in individual pixels in training and test sets.

The division of the data at the slice level was first based on a training/validation/testing ratio of 60/10/30 which is commonly preferred 31-33. Therefore, 43 slices of 18 PDX samples were randomly divided into a training (n=25), validation (n=4) and test datasets (n=14), and 49 slices of 15 RIF-1 samples were randomly divided into a training (n=26), validation (n=5) and test datasets (n=18).

The DL residual neural network (ResNet), a type of CNN that uses residual blocks, achieves state-of-the-art performance in image recognition field. In this study, the architecture of ResNet-18 with small number of filters in each layer was used to predict the hypoxia probability of each pixel, which is shown in **Figure S2**. In details, for each pixel within the tumor region, a 15×15 fixed size sliding window centered on this pixel was used to generate a mp patch from T_2_* map, T_2_W, slope, TTM and AUC images, which was fed into the DL model after z-score normalization for each channel to update the parameters with backward propagation. The binarized average value of the PIMO-positive mask map within this window was encoded to one-hot and used as the label of this patch. The output of the network was used as the classification result to represent the hypoxia probability of each pixel. The final predicted hypoxic habitats could be reconstructed utilizing the location information of each pixel. To guarantee the accuracy of the labels, only samples with similarity score higher than the average scores were involved in the construction of the DL model. The prediction of hypoxia was conducted blindly for each sample.

The CNN models for PDX and RIF-1 tumor models were trained on 138,748 and 154, 873 training patches, respectively, both of which takes around two hours. Using these CNN models, 230 µs was required for the prediction of each patch, which means it took 0.79 s ~ 2.05 s for each tumor slice in this study.

During the training, binary cross entropy was employed as the loss function, while the Adam optimizer was used with an initial learning rate of 0.0001 and decaying by a factor of 0.2 if no improvement of the loss of the validation dataset was seen for 10 epochs. Additionally, augmentation including width/height-shift, horizontal/vertical-flip, rotation and zoom were used to expand the training dataset to improve the ability of the model to generalize. The implementation of this model used the Keras toolkit and Python 3.5. The computations were carried out on a desktop computer with an Intel Xeon E5 CPU and a Nvidia GeForce GTX 1080 GPU with 32GB memory.

### Statistical analyses

Comparison of data between groups was done by using Student's t-test or one-way analysis of variance (ANOVA) followed by multiple comparisons test. Kaplan-Meier was used to estimate survival rates and the log-rank test was used to analyze differences between the groups. P values <0.05 were considered statistically significant.

DSC was calculated to measure the spatial overlap between the predicted hypoxic habitats and PIMO-positive mask quantitatively. The cutoff to binarize the predicted hypoxia probability was determined according to the average optimal value to obtain the largest DSC for each training sample. Give the label of the patch was the binarized average value of the PIMO-positive mask map within this patch, which means the PIMO result is not the real patch-based label, the correlation between the true hypoxia fraction (PIMO-positive fraction in histology) and the predicted hypoxia fraction by the model in the co-registered MRI was analyzed by Pearson correlation coefficient and visualized with regression line rather than identify line (Detailed explanation shown in **Figure S3**).

To measure the predictive ability of the predicted hypoxia fraction in identifying the samples with response to TH-302, area under the receiver operating characteristics curve (AUROC) was used. The optimal cutoff was determined to maximize the Youden's index by balancing the sensitivity and specificity, and the Cox proportional hazards model was used to analyze the prognostic value of the predicted hypoxia fraction.

## Results

### Tumor growth and survival

**Figure 1:**
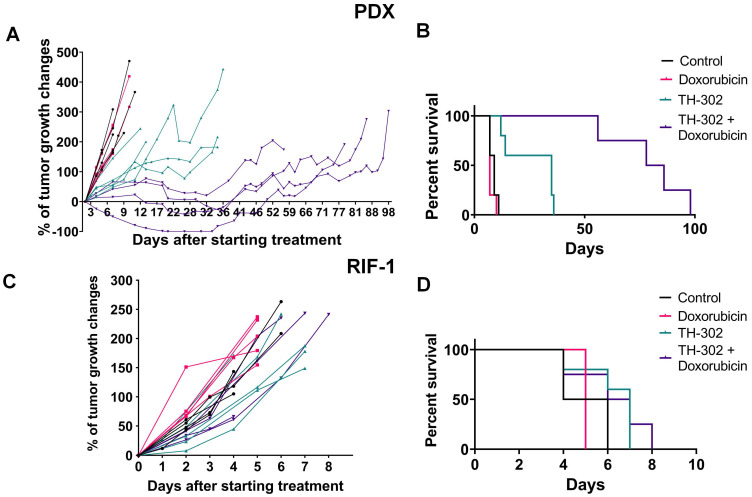
** Tumor growth and survival plots for sarcoma mouse models. A.** Tumor growth changes (%) after starting treatment (day 0) for patient-derived xenograft (PDX) rhabdomyosarcoma model. **B.** Kaplan-Meier plots for PDX shown that monotherapy with TH-302 or with the TH-302 + doxorubicin (Dox) combination increased the overall survival (OS) (p=0.35 Dox *vs* Control; *p=0.019 TH-302 *vs* Control; **p=0.0016 TH-302 *vs* Dox; **p=0.0046 TH-302 + Dox *vs* Control; **p=0.0035 TH-302 + Dox *vs* Dox; **p=0.0051 TH-302 + Dox *vs* TH-302 + Dox). **C.** Tumor growth changes (%) after starting treatment in the radiation-induced fibrosarcoma (RIF-1) model. **D.** Kaplan-Meier plots for RIF-1 shown that there was not significant difference in the OS between groups of treatment (p=0.13 Dox *vs* Control; p=0.08 TH-302 *vs* Control; p=0.06 TH-302 *vs* Dox*;* p=0.16 TH-302 + Dox *vs* Control; p=0.12 TH-302 + Dox *vs* Dox; p=0.73 TH-302 *vs* TH-302 + Dox).

In the rhabdomyosarcoma PDX model, monotherapy with TH-302 or the combination of TH-302 + Dox resulted in reduced tumor growth, while Dox monotherapy was ineffective (**Figure 1A**). Compared to untreated control or the Dox monotherapy arm, the OS significantly increased with both the TH-302 monotherapy (p=0.0019 *vs* Control; p=0.0016 *vs* Dox) and the TH-302 + Dox combination (p=0.0046 *vs* Control; p=0.0035 *vs* Dox). The median survival for control and Dox-treated groups were 9 and 7 days respectively, while it increased to 35 and 82 days when mice were treated with TH-302 monotherapy or TH-302 + Dox combination, respectively (**Figure 1B)**. Further, combination therapy was superior to TH-302 monotherapy in increasing OS, and this may be consistent with concept that TH-302 controls hypoxic habitats while Dox controls the normoxic viable tumor areas 34. In the combination group, 4 of 5 tumors regressed during therapy, but eventually regrew (**Figure 1A**), suggesting that they may have acquired a resistance mechanism during prolonged therapy. All therapies were well tolerated, and mice did not show significant changes in body weight during the therapy course (p-values > 0.05; **Figure S4A**).

In the RIF-1 model, there were no differences in tumor growth between the control mice and mice treated with any therapy (**Figure 1C**). In addition, there were no differences in the OS, with median survivals of 5; 5; 7 and 6.5 days for the Control, Dox, TH-302, or TH-302 + Dox groups, respectively (p-values > 0.05 for all groups;** Figure 1D**). Mouse body weight was not affected during any therapy protocol (p-values >0.05;**Figure S4B**).

### Hypoxia status can determine TH-302 response

**Figure 2:**
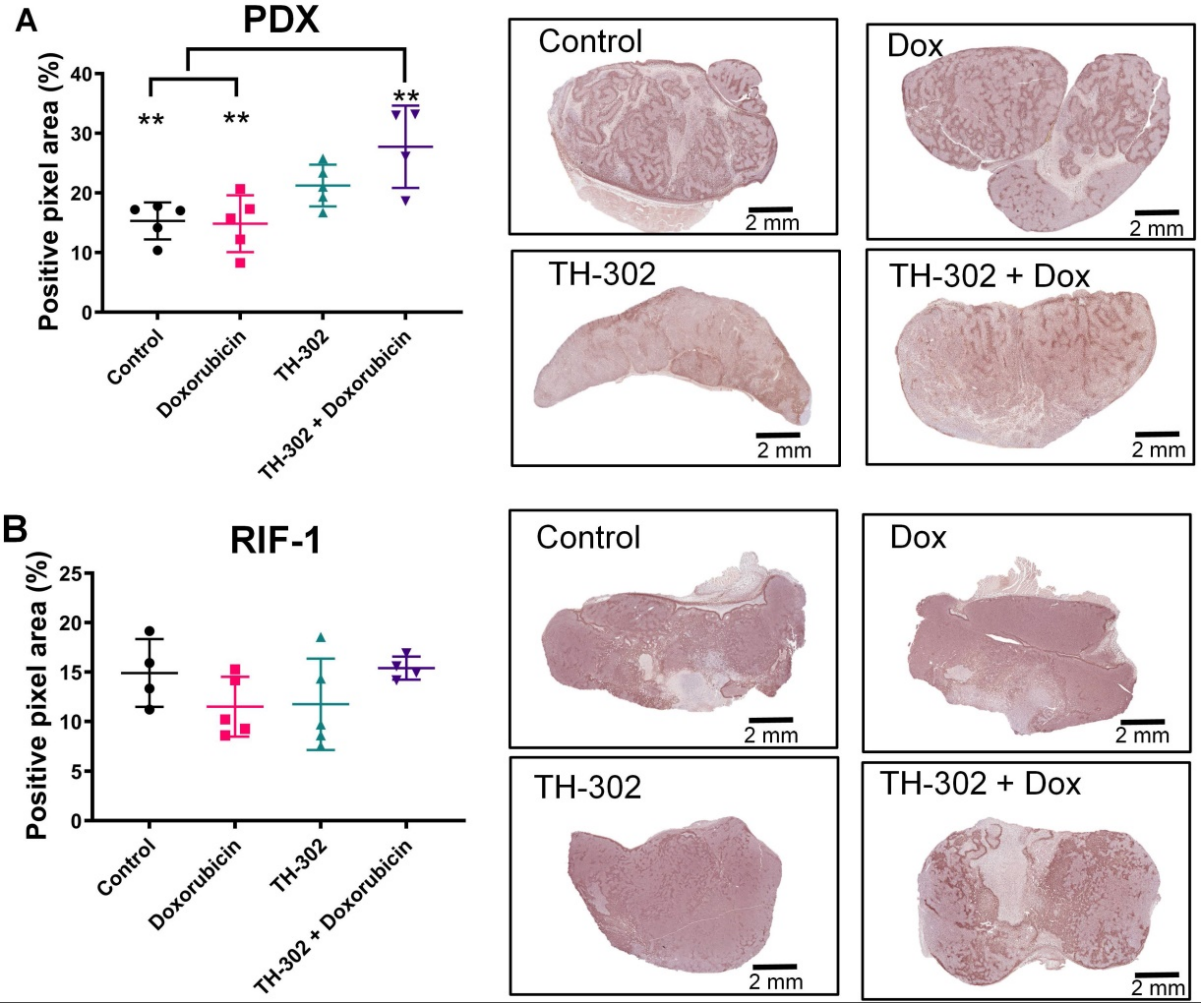
** Quantification and representative images of pimonidazole staining in tumors collected in the last day of therapy at the time of sacrifice. A.** Patient-derived xenograft (PDX) rhabdomyosarcoma model. (p>0.99 Dox *vs* Control; p=0.36 TH-302 *vs* Control; **p=0.006 TH-302 + Dox *vs* Control; p=0.27 TH-302 *vs* Dox; **p=0.005 TH-302 + Dox *vs* Dox; p=0.32 TH-302 *vs* TH-302 + Dox). **B.** Radiation-induced fibrosarcoma (RIF-1) model; (p=0.94 Dox *vs* Control; p>0.99 TH-302 *vs* Control; p>0.99 TH-302 + Dox *vs* Control; p>0.99 TH-302 *vs* Dox; p=0.65 TH-302 + Dox *vs* Dox; p=0.78 TH-302 *vs* TH-302 + Dox). Analysis of variance (ANOVA) followed by Bonferroni multiple comparisons test. Values presented as mean± SD.

**Figure 3:**
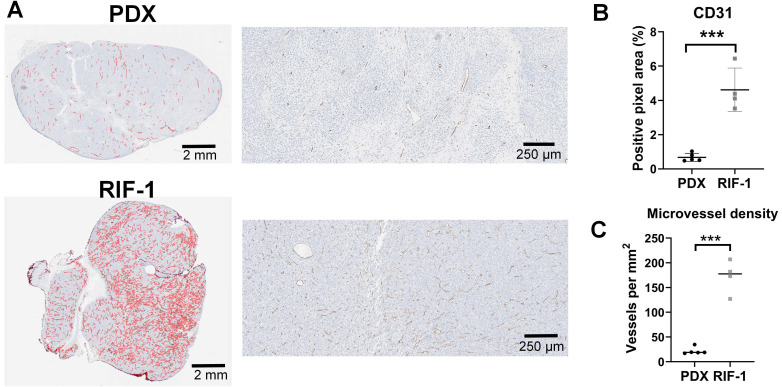
**Cluster of differentiation 31 (CD31) staining for blood vessels. A. Representative images of CD31 staining in patient-derived xenograft (PDX) rhabdomyosarcoma and radiation-induced fibrosarcoma (RIF-1) tumors. B.** Values of CD31-positive area from control groups were compared between PDX and RIF-1 tumors in last day of therapy. ***p=0.0002 by Student's t test to positive pixel area (%); **C.** Microvessel density from control groups was compared between PDX and RIF-1 tumors. ***p<0.0001 by Student's t test.

Surprisingly, the percentage of PIMO-positive pixels was statistically higher in the last day of therapy in the PDX tumors treated with TH-302 + Dox combination when compared with control (p=0.006) and Dox-treated tumors (p=0.005) (**Figure 2A**). The TH-302 monotherapy treated-tumors also appeared to have increased hypoxia, but the results were not significant (p=0.36). For RIF-1 tumors, there was no significant difference in PIMO-positive areas between control and any of the therapy groups (p>0.05; **Figure 2B**). While the proportion of positive pixels was similar for both tumor types under control conditions, these values cannot be directly compared as the two tumor types are physiologically distinct with respect to cell density, etc. For example, tumors were also stained with a blood vessel marker CD31. RIF-1 tumors were much more vascularized than PDX tumors, showing significantly higher CD31 staining (p=0.0002) and microvessel density (p<0.0001) (**Figure 3**), suggesting that a well-perfused and well-oxygenated tumor environment can be contributing to the non-response to TH-302 in the RIF-1 tumors.

### RIF-1 resistance to TH-302 therapy is due to lack of hypoxia

Although unlikely, it is possible that the mechanisms responsible for TH-302 resistance in the RIF-1 model involved adaptive immunity. The resistant RIF-1 model is syngeneic (RIF-1 cells inoculated into C3H mice), but the sensitive PDX model is immune compromised (human cells inoculated into SCID/SHO athymic mice). To evaluate this, we grew RIF-1 tumors in NSG immunodeficient mice. As shown in**Figure S5,** TH-302 therapy was also not effective in this immunocompromised NSG model, showing similar results as observed for the immunocompetent C3H model. These results indicate that TH-302 resistance is not mediated by an adaptive immune response. However, it is important to note that NSG mice retain elements of the innate immune system including macrophages and neutrophils 35, which could participate in the overall response to therapy, in both immunocompetent and immunocompromised mice.

**Figure 4:**
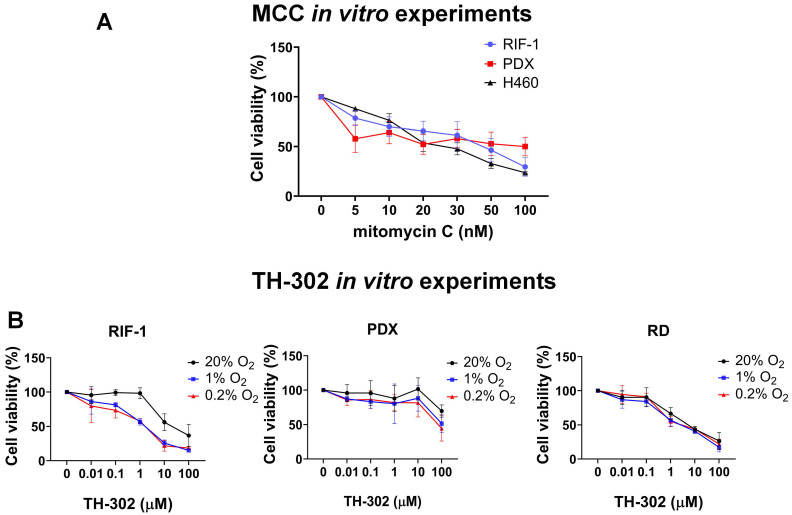
***In vitro* experiments to test cell viability (%). A.** Dose-dependent mitomycin C (MCC) treatment for 72 h. Only p-values <0.05 are shown. P-values for radiation-induced fibrosarcoma (RIF-1) cells: **p=0.007 for Control (C) (0 nM) *vs* 50 nM MCC; ***p=0.0007 for C *vs* 100 nM MCC. P-values for PDX cells: *p=0.04 for C *vs* 100 nM MCC. P-values for H460 cells: ***p<0.001 C *vs* ≥ 20 nM MCC. **B.** Dose-dependent TH-302 treatment under normoxic (20% O_2_) or hypoxic conditions (1% O_2_ and 0.2% O_2_). Only p-values <0.05 are shown. P-values for RIF-1 cells: ***p<0.001 for Control (C) (0 µM) *vs* ≥ 10 µM at 20% O_2_, and ≥ 1 µM at 1% O_2_ and 0.2% O_2_. p-values for patient-derived xenograft (PDX) rhabdomyosarcoma cells: **p<0.01 for C *vs* 100 µM at 1% O_2_ and 0.2% O_2_. p-values for RD cells: **p<0.005 for C *vs* ≥ 1 µM at 20% O_2_, 1% O_2_ and 0.2% O_2_. Analysis of variance (ANOVA) followed by Dunnett's multiple comparisons test.

Another possible source of resistance could be a biochemical resistance to alkylating agents, e.g. through enhanced DNA repair processes. To investigate this, we tested if RIF-1 cells *in vitro* were affected by a DNA cross-linking agent MCC. We used MCC because Br-IPM is too hydrophilic to diffuse at significant rates across the plasma membrane and it is less cytotoxic when added to extracellular medium compared to when it is generated intracellularly from the prodrug 36. As shown in **Figure 4**, RIF-1 cell viability was highly sensitive to MCC, indicating that these cells can respond to alkylating agents, such as Br-IPM. H460 cells (human lung cancer cell line) were used as positive control as a known MCC sensitive line.

Finally, to check if controlled conditions of hypoxia would improve sensitivity to TH-302 in the RIF-1 cells, we tested TH-302 therapy *in vitro* under hypoxia and normoxia. These experiments demonstrated that RIF-1 cells were highly sensitive to TH-302 under hypoxic conditions. There was a concentration-dependent response to TH-302 under hypoxia, while only higher concentrations were effective under normoxia in both the RIF-1 and PDX cells. The human rhabdomyosarcoma cell line (RD cells) were used as positive control 37 and showed sensitivity to TH-302 in doses > 1 µM under normoxia and hypoxia conditions (**Figure 4)**.

### Noninvasive measurement of hypoxia in MR imaging

The above data indicates that RIF-1 cells can respond to TH-302, but only at hypoxic conditions. This emphasizes the importance of identifying tumor hypoxia at the stage of treatment planning in order to predict response.

In addition, increase of PIMO staining observed in TH-302 and TH-302 + Dox treated tumors collected on the last day of therapy raises the question whether there were changes in hypoxia during therapy that could be measured with longitudinal imaging.

Thus, we used the co-registered mpMRI maps and PIMO stained histology to train CNN models to identify hypoxia in MR imaging. Using these models, we were able to non-invasively identify and quantify hypoxia pre-therapy and longitudinally during therapy with MRI.

For the PDX tumor model, 57 slices were co-registered, and the average of DSC was 0.92 ± 0.02 (median = 0.93), while for the RIF-1 tumor model, 63 slices were co-registered with an average DSC of 0.93 ± 0.02 (median = 0.93) as shown in **Table S1**. Subsequently, only samples with greater than average similarity scores of ≥ 0.92 were used to develop the CNN models.

**Figure 5:**
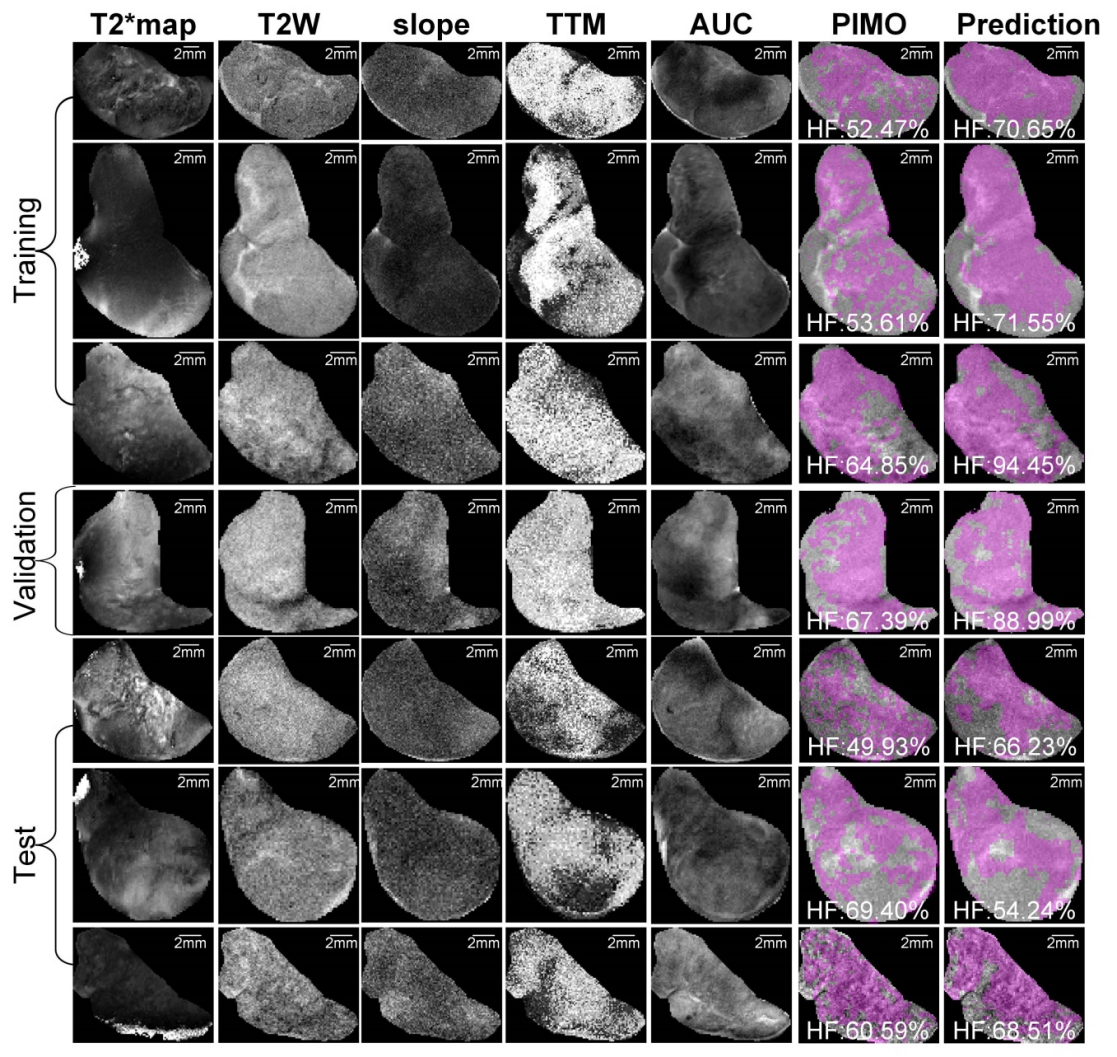
**Representative samples of training, validation and test datasets for Patient-derived xenograft (PDX) rhabdomyosarcoma model.** Images shows multiparametric MRI maps (T2* map, T2-weighted image (T2W) and slope, time to maximum (TTM) and area under the curve (AUC) from dynamic contrast enhanced (DCE) MRI), co-registered pimonidazole stained histology slice (PIMO), and predicted hypoxia fraction (cutoff: 0.4). Note. HF represents hypoxia fraction.

**Figure 6:**
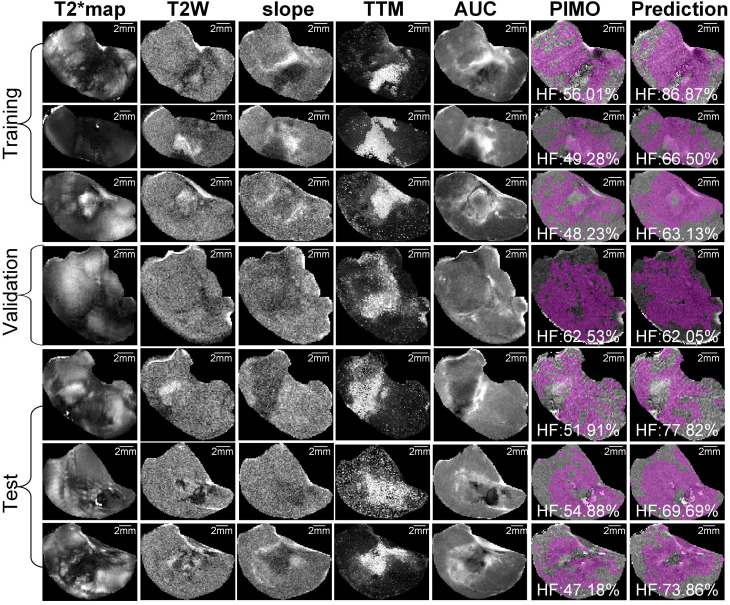
**Representative samples of training, validation and test datasets for radiation-induced fibrosarcoma (RIF-1) tumors.** Images shows multiparametric MRI maps (T2* map, T2-weighted image (T2W) and slope, time to maximum (TTM) and area under the curve (AUC) from dynamic contrast enhanced (DCE) MRI), co-registered pimonidazole stained histology slice (PIMO), and predicted hypoxia fraction (cutoff: 0.4). Note. HF represents hypoxia fraction.

Representative mpMRI maps, co-registered PIMO stained histology slice and the predicted hypoxia fraction of PDX and RIF-1 tumor models from training, validation and test datasets are shown in **Figures 5 and 6**, respectively. Additional samples for each tumor model are shown in **Figures S6 and S7**.

**Figure 7:**
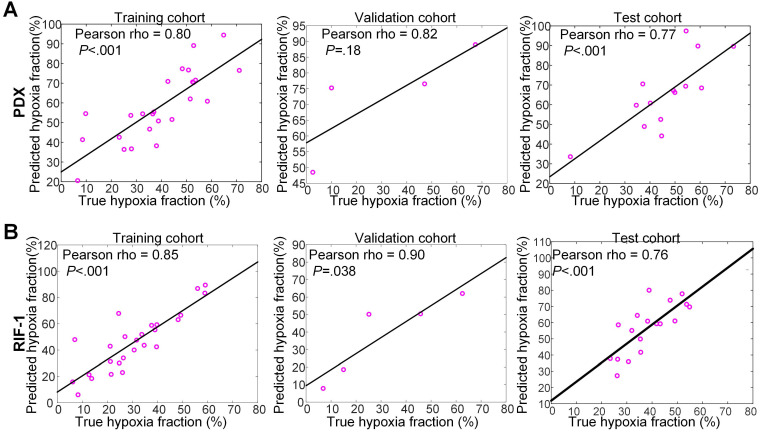
**Correlation between true hypoxia fraction (from pimonidazole stained histology) and predicted hypoxia fraction (from multiparametric MRI).** Plots of training, validation and test cohorts for patient-derived xenograft (PDX) rhabdomyosarcoma **(A)** and radiation-induced fibrosarcoma (RIF-1) **(B)** tumors. N.B. The non-zero intercept is a consequence of using patch-based labeling (see Figure S3).

For PDX tumors, strong correlations of 0.80 (p<0.001), 0.82 (p=0.18), and 0.77 (p<0.001) were found between true hypoxia fraction and predicted hypoxia fraction in the training, validation, and test cohorts, respectively. For the RIF-1 tumors, the correlations were also as strong as 0.85 (p<0.001), 0.90 (p=0.038) and 0.76 (p<0.001) in the training, validation and test cohorts, respectively. Detailed plots are provided in **Figure 7** and detailed quantitative metrics for each slice are shown in **Table S2**.

### Hypoxia status prior to therapy can determine TH-302 response

**Figure 8:**
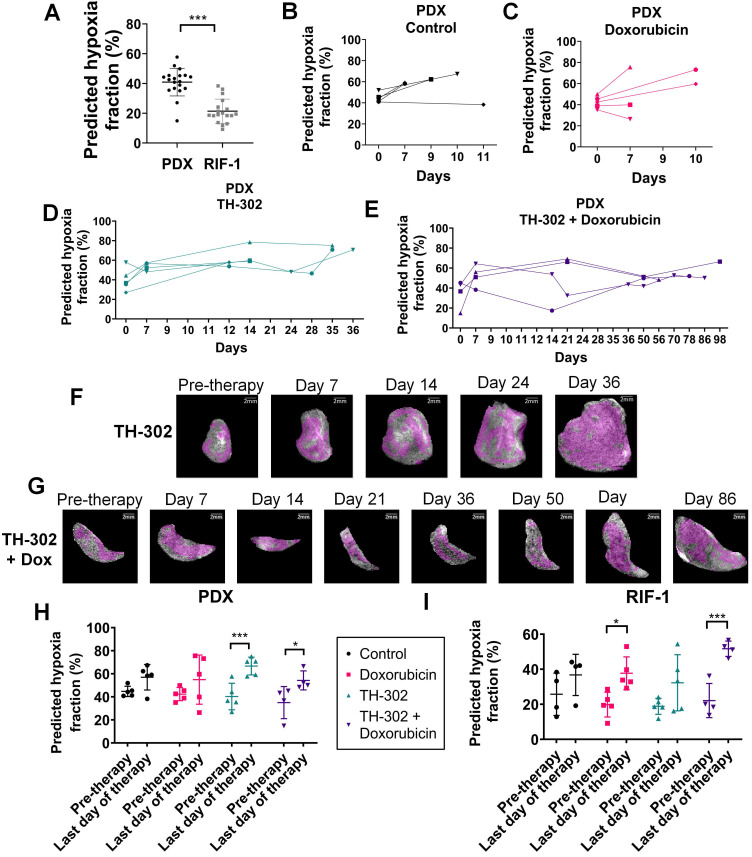
** Predicted hypoxia fraction calculated in pre-therapy MR imaging. A.** Comparison between predicted hypoxia fraction in pre-therapy MR imaging for patient-derived xenograft (PDX) rhabdomyosarcoma and radiation-induced fibrosarcoma (RIF-1) tumor models; ***p<0.0001, Student's t-test. **B-E.** Longitudinal measurements of predicted hypoxia fraction in MRI for the PDX tumor model for control group **(B)**, Doxorubicin (Dox) treated group **(C)**, TH-302 treated group **(D)** and TH-302 + Dox treated group **(E)**. **F-G.** Representative images of changes in predicted hypoxia fraction (in magenta) during therapy for a TH-302-treated tumor in (**F)** and for a TH-302 + Dox-treated tumor in **(G)**;** H-I.** Comparison of predicted hypoxia fraction in pre-therapy and last day of therapy between groups of therapy for PDX tumor model (Pre-therapy *vs* last day: p=0.15 Control; p=0.14 Dox; ***p=0.0009 TH-302; *p=0.02 TH-302 + Dox) in **(H)**; and for RIF-1 tumor model (Pre-therapy *vs* last day: p=0.35 Control; *p=0.01 Dox; p=0.10 TH-302; ***p=0.0009 TH-302 + Dox) in **(I)**. Analysis of variance (ANOVA) followed by the Bonferroni test.

Comparison of hypoxic status at pre-therapy between tumor models confirmed that predicted hypoxia portion was significantly lower in RIF-1 than PDX tumors, which is consistent with the response to TH-302 in the PDX model, and resistance in the RIF-1 model (**Figure 8A**).

Interestingly, survival of PDX mice treated with TH-302 monotherapy increased to 35 days, however there was one mouse that did not respond to TH-302, reaching the limit tumor volume 12 days after starting therapy. Subsequent to the above CNN analyses, we observed that this mouse showed the lowest level of predicted hypoxia by pre-therapy MRI among this group, which is consistent with the non-response to TH-302.

To test if levels of hypoxia prior to therapy could predict response to TH-302, we analyzed all mice treated with TH-302 and TH-302 + Dox regardless of PDX or RIF-1 tumor models, consistent with the SARC21 (NCT01440088) clinical trial wherein STS patients were treated regardless of histotype. Here, we compared the CNN-generated predicted fraction of pre-therapy hypoxia to the TH-302 response with a median survival cutoff of 14 days. This generated an AUROC of 0.89 (95%CI: 0.72, 1.00, p=0.005), and a C-index of 0.73 (95%CI: 0.57, 0.88, p=0.004) in predicting the survival >14 days. The optimal cutoff of 25.60% was obtained based on the maximum Youden's index in the receiver operating characteristics (ROC) curve to identify the mice which are more likely to respond to TH-302. Using this cutoff, mice were stratified into high- and low-hypoxia fraction groups. The mice within the high-hypoxia fraction group had longer survival with a median value of 35 (interquartile range (IQR): 14-75) days versus 7 (IQR: 6-7) days for the low-hypoxia fraction group. Using Cox proportional hazards regression analysis, the binarized predicted hypoxia fraction with this cutoff was identified as significant prognostic factor with the hazard ratio (HR) of 0.27 (95%CI: 0.090, 0.83, p=0.022) in survival prediction.

### Temporal evolution of hypoxic habitats

**Figures 8B-G** present longitudinal measurements of predicted hypoxia fraction for the PDX tumor model, which showed paradoxically high levels of hypoxia by PIMO following a course of successful therapy (see **Figure 1A**). An increase in predicted hypoxia after day 7 for most of the tumors regardless the therapy was observed **(Figures 8B-E**). However, for tumors treated with TH-302 or with TH-302 + Dox, the hypoxic fraction was decreased or controlled during the course of the therapies (**Figures 8D-E**). More specifically for the TH-302 group, one mouse showed a decrease of 9.5% at day 7, while it was decreased or controlled in other 3 mice after day 14. For the mouse that did not respond to TH-302, there was an increase of 31% of hypoxia from pre-therapy to day 12 (diamond symbol in **Figure 8D**). All mice treated with TH-302 + Dox showed a decrease in hypoxia at different time points during therapy. In the first measurement after starting therapy, hypoxic fraction decreased in one mouse but increased in the other 3 mice. It was reduced at day 14 for one mouse, and after day 50 for the other 2 mice, however, eventually an increased in hypoxia was observed in this group, as the tumors grew, and the resistance to TH-302 emerged (**Figure 8E**).

When comparing values of predicted hypoxia fraction from pre-therapy with the final measurement taken before sacrifice, more hypoxia was observed in the last day of therapy for all groups of PDX tumors. Surprisingly, this increase in hypoxia was statistically significant in the tumors treated with TH-302 monotherapy (p=0.0009) and with the TH-302 + Dox combination (p=0.02), as shown in **Figure 8H**. These results are consistent with PIMO staining observed at the histology collected before sacrifice, which showed higher levels in TH-302 and TH-302 + Dox treated groups than control and Dox-treated groups (cf. **Figure 2**). For RIF-1 tumors, the predicted hypoxia fractions were also higher on the last day of therapy, and it was statistically significant for the Dox-treated tumors (p=0.01) and tumors treated with the TH-302 + Dox combination (p=0.0009), shown in **Figure 8I**. As discussed below, this increase in hypoxic volume fraction may be due to the tumors' increased volume relative to its perfused volume, further emphasizing the need to measure hypoxic fractions longitudinally. Longitudinal measurements of predicted hypoxia fraction for each therapy group of the RIF-1 tumor model are shown in **Figure S8.**

For the PDX model, we also noted that the percentage of cells stained with the apoptosis marker CC3 in the TH-302 treated tumors was significantly lower than control and Dox-treated tumors in the last day of therapy (p<0.05; **Figure S9A**), while phospho gamma-H2AX did not show significant changes (p>0.05; **Figure S9B**), which corroborated the hypothesis that, at the time of sacrifice, tumors were therapy resistant.

## Discussion

In this study, we used the HAP TH-302 to target hypoxia in sarcoma mouse models, and we developed a deeply learned MRI-based method to predict hypoxia prior to and longitudinally during therapy. We showed that TH-302 monotherapy or in combination with Dox was able to delay tumor growth and increase survival in a PDX model of rhabdomyosarcoma, while a syngeneic RIF-1 fibrosarcoma model was resistant to TH-302.

Notably, Dox monotherapy was not effective in either PDX or RIF-1 tumor models. In the PDX, a possible explanation would be the high levels of hypoxia arising from poor perfusion in these tumors. RIF-1 tumors have shown to be vascularized and a response to Dox would have been expected. This resistance is not intrinsic to the RIF-1 cells, as we showed in their *in vitro* responsiveness to TH-302 or MCC. Resistance of RIF-1 tumors to Dox *in vivo* has been observed by others 38. As it does not appear to be perfusion-mediated, we speculate that resistance may be due to 1) a smaller fraction of cells in the cell cycle; 2) stromal protection; or 3) elevated interstitial fluid pressure, all of which are known mechanisms to confer resistance to Dox.

Hypoxic status has been associated with TH-302 response in several pre-clinical models 19, 34, 39. However, resistance was observed in hypoxic tumors 40, as TH-302 efficacy is also dependent on other conditions, such as prodrug-activating reductases, intrinsic sensitivity to the drug warhead and DNA repair status 41. Here, we explored different mechanisms that could be contributing to TH-302 resistance in RIF-1 model and showed that hypoxia status may be the causal effect. Pre-therapy MR imaging showed that RIF-1 tumors are less hypoxic than PDX tumors. In fact, RIF-1 tumors are known to present a small fraction of radiobiologically hypoxic cells 42, 43.

This emphasizes the importance of knowing the hypoxia status in order to individualize and hence optimize therapies using HAPs. As mentioned before, although TH-302 monotherapy or in combination with chemo- or radiotherapy have been showing promising results in pre-clinical studies, there has not been much progress in clinical studies, with its failure in improving OS in phase III clinical trials in advanced pancreatic cancer (MAESTRO; NCT01746979) or soft tissue sarcoma (TH CR-406/SARC021). The reasons for this failure are multifaceted but neither study stratified patients based on their tumor hypoxia-status 14. Notably, in the MAESTRO trial, the combination of TH-302 + gemcitabine was shown to be efficacious in increasing PFS (p=0.004) and the objective response rate (ORR, p=0.009), but the primary endpoint of OS was not significantly different (p=0.059) when compared to the gemcitabine + placebo group 44. In this context, it can be proposed that a biomarker-stratified study design, with upfront assessment of hypoxia, would increase the chance to achieve clinical benefit from HAPs, with fewer trial patients needed 14.

Multiple approaches have been used for hypoxia detection 45, but priority must be given to imaging methods. MRI methods such as Blood Oxygen Level Dependent (BOLD) 46 or Oxygen Enhanced (OE)-MRI 47, designed to provide insight into blood and tissue oxygenation respectively, have been shown to correlate with hypoxia *ex vivo* 48. The intrinsic low sensitivity and other confounding factors 49 may however limit the wide application of these functional techniques. Our imaging approach can easily be translated to the clinic, as DCE-MRI is routinely used and is reproducible, which allow not only pre-therapy measurement, but also longitudinal assessment of hypoxia to follow therapy response.

However, it is important to consider the safety of longitudinal imaging that requires administration of gadolinium (Gd)-based contrast agents (GBCA), especially in sarcoma that shown high prevalence in children. Although macrocyclic chelates as gadobutrol used in this study have shown to be safe and not leach Gd or induce nephrogenic systemic fibrosis, it has been demonstrated that a variability of GBCA classes, but especially the linear chelates, can cause small fraction of Gd retention in human tissues. Thus, repeated administration of GBCA must be planned carefully, especially in pediatric patients, as long-term effects of Gd tissue accumulation have not been fully characterized 50. Although our pre-clinical study performed longitudinal measurements repeatedly during therapy, high frequency DCE-MR scans are generally not acquired clinically. Based on our preclinical observations, a pre-therapy imaging session is important to define the therapy based on the extent of hypoxia, and two or three additional measurements along the treatment would be informative of HAPs therapy response. Additional studies are necessary to establish the optimal time points when MRI should be considered for maximal impact to clinical care.

In addition, it is worth emphasizing that our CNN-based imaging approach provides spatial information of the heterogeneous distribution of hypoxia, and it reflects both acute and chronic hypoxia, as PIMO histology was used as the true hypoxia fraction to train the model 51. Distinguishing between acute and chronic hypoxia would be interesting to evaluate if longitudinal assessment of acute hypoxia also has a true value in the therapy choice and monitoring. In the past, we and others have used time-dependent changes in the T_2_* signal to identify temporal changes in HbO_2_ status as a surrogate for tissue pO_2_ 52, and future work to combine the CNN maps with temporal T_2_* are being explored.

This spatial information is especially important to optimize combination therapy regimens considering the tumor evolutionary dynamics. Here, we showed that combination of TH-302 + Dox was much more effective than TH-302 monotherapy in the PDX model, showing an initial suppression of hypoxia; however, both therapeutic regimes lead to resistance with prolonged treatment, with an increase in the hypoxia fraction being observed later on during the therapies. A rationale for using a combination of TH-302 and Dox is to target two different populations within the heterogeneous tumor microenvironment, which would lead to complete tumor eradication or long-term control compared to monotherapies that affect only the normoxic or hypoxic adapted populations. However, optimal therapy efficiency is highly dependent on identifying the right timing and administration sequence of combination therapies 11. In our study, Dox was given once a week and TH-302 was given daily, 5 days a week.

Thus, our data suggest that with the continued use of TH-302 and Dox, a state of dynamic equilibrium between hypoxic and viable normoxic tumor cell populations was lost, with TH-302 not being able to continue controlling the population in the hypoxic habitat. Therapy-sensitive and resistant cell types constantly compete into the tumor microenvironment; however, this equilibrium can change with prolonged treatment, leading to emergence of a resistant population. The residual cell population that persisted after the several rounds of therapy is likely to have greater intrinsic or environmental resistance, and will continue to survive with the continued use of the same therapeutic regimen 53, 54. A different study reached a similar conclusion with an epidermal growth factor receptor (EGFR)- targeted agent with a mathematical model showing that a longer time under TH-302 therapy without the targeted inhibitor erlotinib allowed the EGFR- sensitive cell population to expand drastically due to TH-302 resistance 11. Indeed, it has been observed clinically that resistance to different individual therapies that are used in combination can emerge asynchronously 55.

Following tumor hypoxia longitudinally in the clinic using imaging approaches as developed in this study, would allow an optimal time planning for switching drugs, avoiding unnecessary doses or drugs and could predict future response or resistance to therapy. In addition, it could guide adaptive therapy, which adjusts the time course of therapy to turn it on and off accordingly, to maintain the sensitive cells populations that will compete and continue to suppress the resistant population 53. Unlike simple whole-tumor metrics, the spatial insight available from the method may provide a detailed picture of this evolutionary balance.

Our results show that different responses to TH-302 in PDX rhabdomyosarcoma and RIF-1 fibrosarcoma seems to be associated with status of tumor hypoxia. A major limitation and unknown remaining from this study is the mechanism underlying the resistance that emerges in PDX tumors over the course of therapy. RIF-1 tumors are intrinsically resistant due to low hypoxic volumes. However, it is notable that RIF-1 tumors get very large and hypoxic, they are still resistant. We speculate that, at the point, the tumors were too large to control at the given dose. Notably, the PDX was originally sensitive but developed physiological resistance during TH-302 monotherapy or in combination with Dox, and this resistance was not related to a reduction in hypoxic volumes. Another limitation is the small number of the cohorts and most tumors in the training cohorts has hypoxia fraction larger than 20%, so the models could not achieve good performance in tumors with small hypoxia fraction (<10%). We will improve the DL models with accruing more data in the future. Third, all sequences in this study were obtained with a slice thickness of 1 mm, the performance of the models may be decreased for clinical images with larger slice thickness due to poor image quality and larger partial volume effect. However, this can be mitigated by differences in the sizes of tumors, with sarcomas being 100-1000 times larger in humans compared to mice.

In conclusion, non-invasive MR imaging to identify hypoxia prior to therapy can presage the initial responsiveness to TH-302 and longitudinally monitor its antitumor effect. Specifically, hypoxia imaging developed here can be applied in further studies, where cycle treatments between TH-302 and Dox can be optimized depending on the extent of hypoxic habitats to avoid or delay the emergence of resistance.

## Supplementary Material

Supplementary figures and tables.

## Abbreviations

ABC: avidin biotin complex
abs: Absorbance
ANOVA: analysis of variance
AUC: area under the time-series curve
AUROC: area under the receiver operating characteristics curve
Br-IPM: bromo-ifosfamide mustard
CC3: Cleaved Caspase-3
CD31: Cluster of differentiation 31
CNN: convolutional neural network
CSTN: Childhood Solid Tumor Network
DCE: dynamic contrast enhanced
DL: deep-learning
Dox: doxorubicin
DSC: Dice similarity coefficient
DWI: diffusion-weighted imaging
EGFR: Epidermal growth factor receptor
FBS: fetal bovine serum
FOV: field of view
H460: human lung cancer cell line
GBCA: gadolinium-based contrast agent
Gd: gadolinium
HAPs: hypoxia-activated prodrugs
HBSS: Hanks' Balanced Salt Solution
HEPES: N-2-hydroxyethylpiperazine-N-ethanesulfonic acid
HR: hazard ratio
IACUC: Institutional Animal Care and Use Committee
IHC: immunohistochemistry
IP: intraperitoneal
IQR: interquartile range
IRB: Institutional Review Board
IV: intravenous
MCC: mitomycin C
MGE: multi gradient echo
mp: multiparametric
MRI: magnetic resonance imaging
MSME: multi slice multiecho
NSG: NOD scid gamma mouse
ORR: objective response rate
OS: overall survival
PDX: patient-derived xenograft
PET: positron emission tomography
PIMO: pimonidazole
PFS: progression free survival
P/S: penicillin/streptomycin
RD: human rhabdomyosarcoma cell line
ResNet: Deep residual neural network
RIF-1: radiation-induced fibrosarcoma cell line
ROC: receiver operating characteristics
RPMI: Roswell Park Memorial Institute
RT: room temperature
SCID: severe combined immunodeficiency
SDS: sodium dodecyl sulfate
SHO: SCID Hairless Outbread
STR: short tandem repeat
STS: soft tissue sarcoma
T_2_W: T2-weighted MRI
TE: echo time
TR: repetition time
TTM: time to maximum
