# Supplementary material for: Deep-learning and MR images to target hypoxic habitats with evofosfamide in preclinical models of sarcoma
Source: Theranostics. 2021 Mar 11;11(11):5313–29. doi: 10.7150/thno.56595 (PMC8039958; doi:10.7150/thno.56595)
Supplement: Supplementary file 1 — Supplementary figures and tables. [file thnov11p5313s1.zip › Supplemental Figures.pdf]

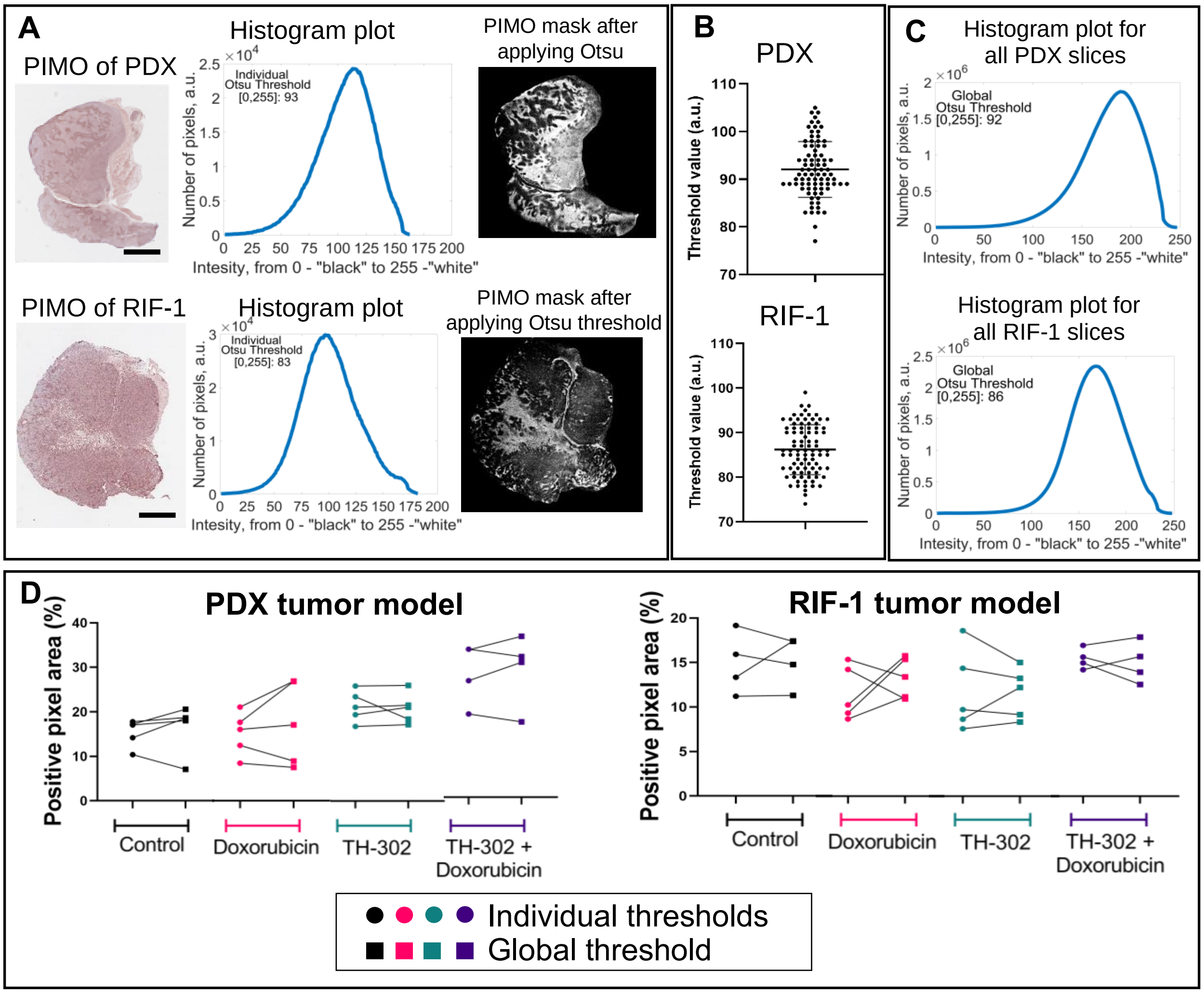


**Figure S1. Thresholding method (global or individual) did not affect calculation of pimonidazole (PIMO)-positive pixels. A.** Representative histology slices stained with PIMO (scale bar = 3 mm); histogram plots showing the distribution of PIMO pixels intensity for individual histology slices to calculate threshold; and masked images with individual thresholds for patient-derived xenograft (PDX) rhabdomyosarcoma and radiation-induced fibrosarcoma (RIF-1) tumors. **B.** Distribution of individual thresholds values for PDX and RIF-1 histology slices (mean for all PDX slices was 92.06 ± 5.85 and for RIF-1 slice was 86.17 ± 5.58) **C.** Histogram plots showing the distribution of PIMO pixels intensity for all histology slices to calculate a global threshold for PDX and RIF-1 tumors. **D.** Comparison of positive-pixel area (%) per groups when using individual thresholds for each slice to identify PIMO-positive areas (represented by circle) or a global threshold for all slices (represented by squares). There was not significantly difference in the percentage of PIMO-positive area in each group when using individual or global thresholding methods; $p>0.05$ by Paired t test. PDX tumor model (Control: $p=0.38$, mean of difference = 1.28; Doxorubicin (Dox): $p=0.37$, mean of difference = 2.27; TH-302: $p=0.70$, mean of difference = -0.47, TH-302 + Dox: $p=0.58$, mean of difference = 0.9), RIF-1 tumor model (Control: $p=0.83$, mean of difference = 0.30; Dox: $p=0.41$, mean of difference = 1.74; TH-302: $p=0.87$, mean of difference = -0.19, TH-302 + Dox: $p=0.69$, mean of difference = -0.42).

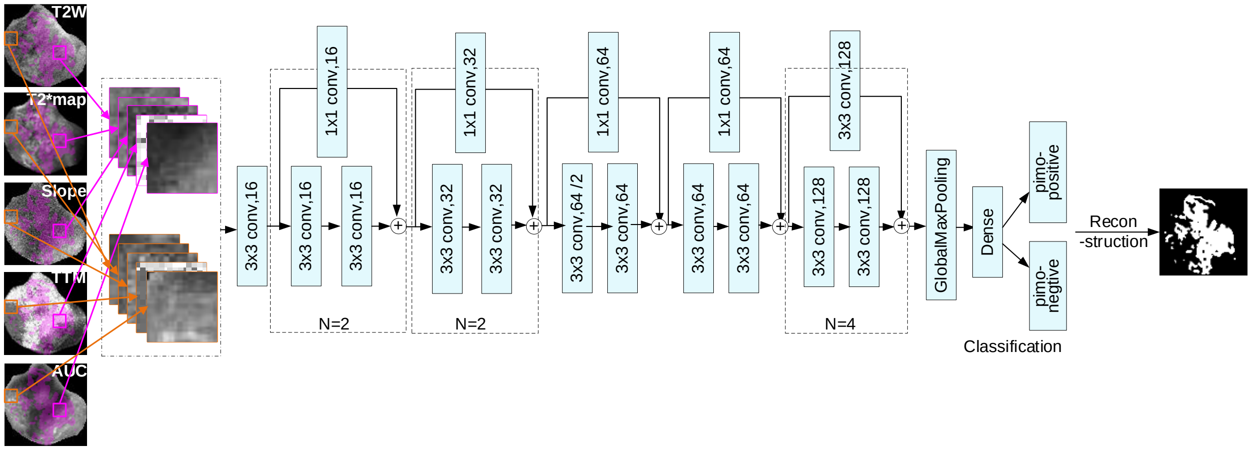


**Figure S2. Schematic representation of the architecture of ResNet-18 used in this study.** $T_2$* map, $T_2$-weighted image ($T_2$W) and slope, time to maximum (TTM) and area under the curve (AUC) from dynamic contrast enhanced (DCE) MRI.

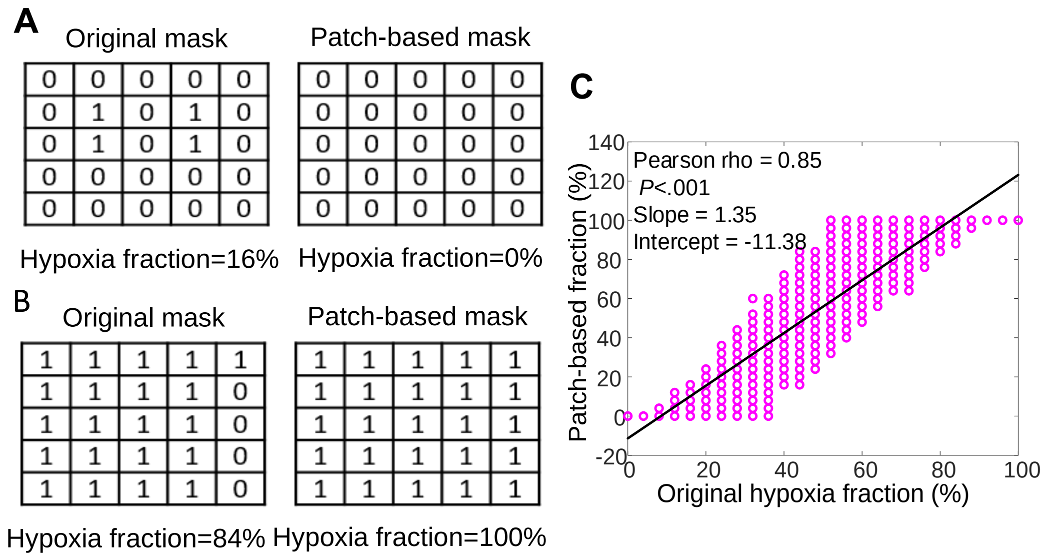


**Figure S3.** The comparison between the original mask and the patch-based mask. A 5x5 size image with the patch size of 3x3 size was used as an example. **A.** represents the situation that the PIMO only includes some isolated hypoxia pixels. The hypoxia fraction of the original PIMO mask is 16%, while 0% for the patch-based mask. **B.** represents the situation that though not all the pixels are PIMO positive (84%), the hypoxia fraction of the patch-based mask is 100%. **C.** The correlation between original hypoxia fraction and patch-based hypoxia fraction for all the simulated 33,554,432 (2^25) combination within in this 5x5 size image. The regression line has a non-zero intercept and a slope larger than 1. Therefore, the intercept and slope of the regression line between the original PIMO true hypoxia fraction and patch-based hypoxia fraction are not 0 and 1.

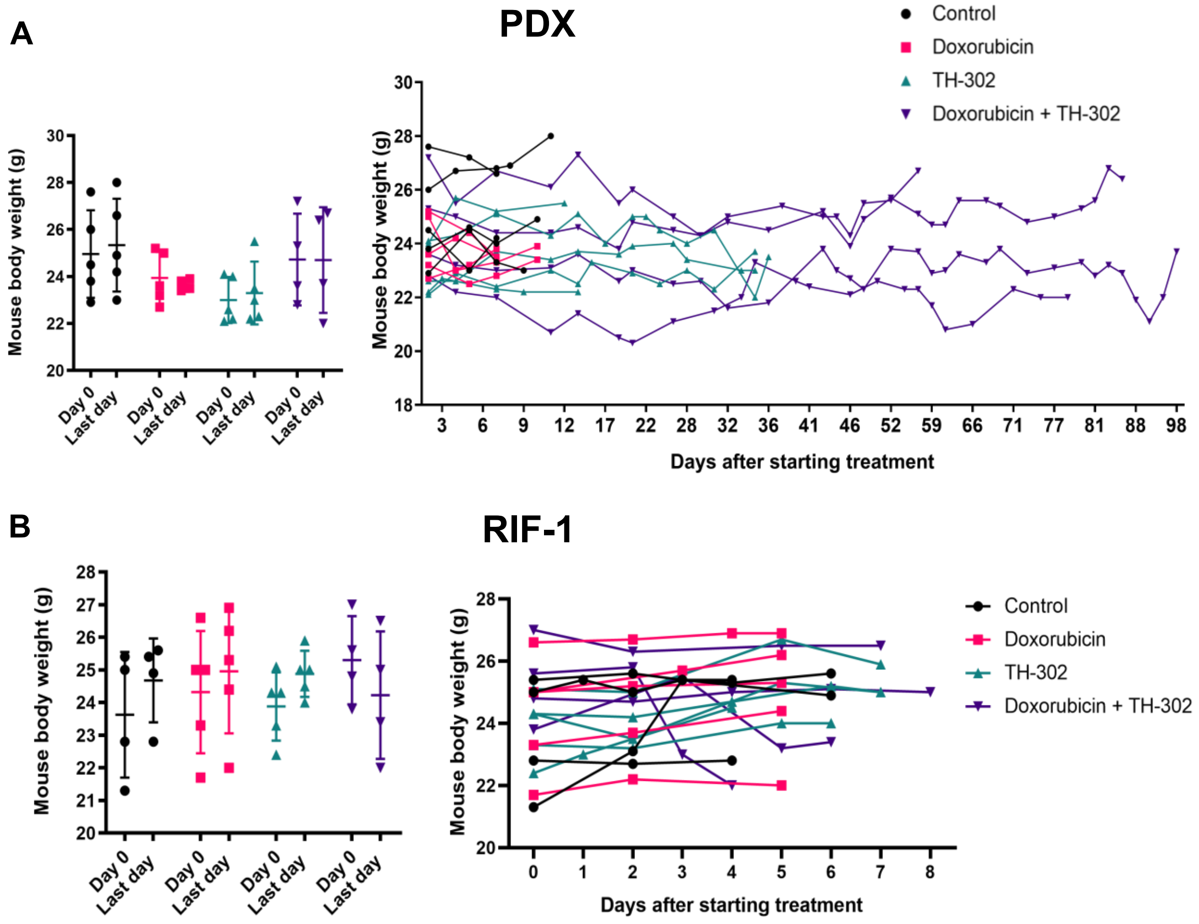


**Figure S4. Body weight during therapy and comparison between day 0 and last day of therapy. A.** Patient-derived xenograft (PDX) rhabdomyosarcoma model. p-values of day 0 *vs* last day, p=0.76 Control; p=0.54 Doxorubicin (Dox); p=0.69 TH-302; p>0.98 TH-302 + Dox. **B.** Radiation-induced fibrosarcoma cell line (RIF-1) model. p-values of day 0 *vs* last day, p=0.39 Control; p=0.60 Dox; p=0.11 TH-302; p>0.39 TH-302 + Dox.

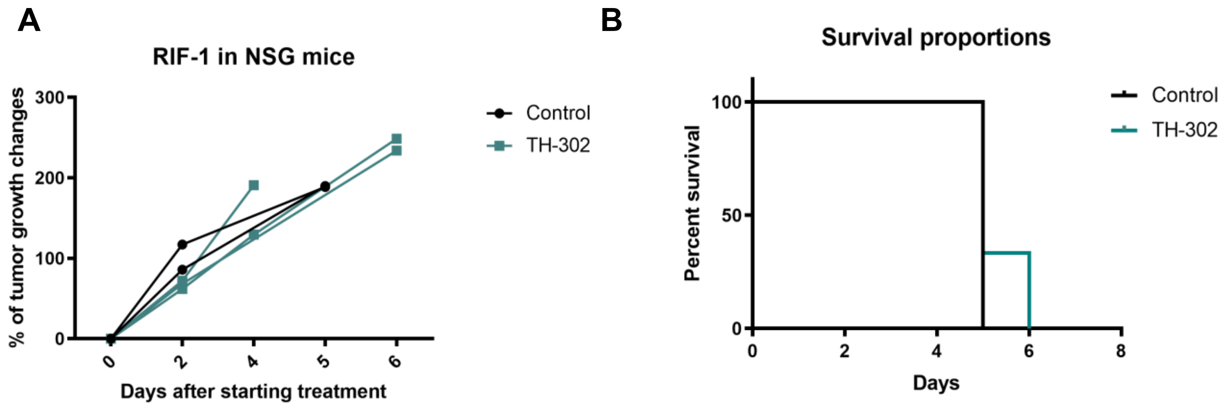


**Figure S5. Tumor growth and survival plots for NSG (NOD scid gamma) immunodeficient mice inoculated with RIF-1 cells. A.** Tumor growth changes (%) after starting treatment. No differences were observed between groups. **B.** Kaplan-Meier plots show that there is no significantly difference in the OS between groups of treatment (p=0.41).

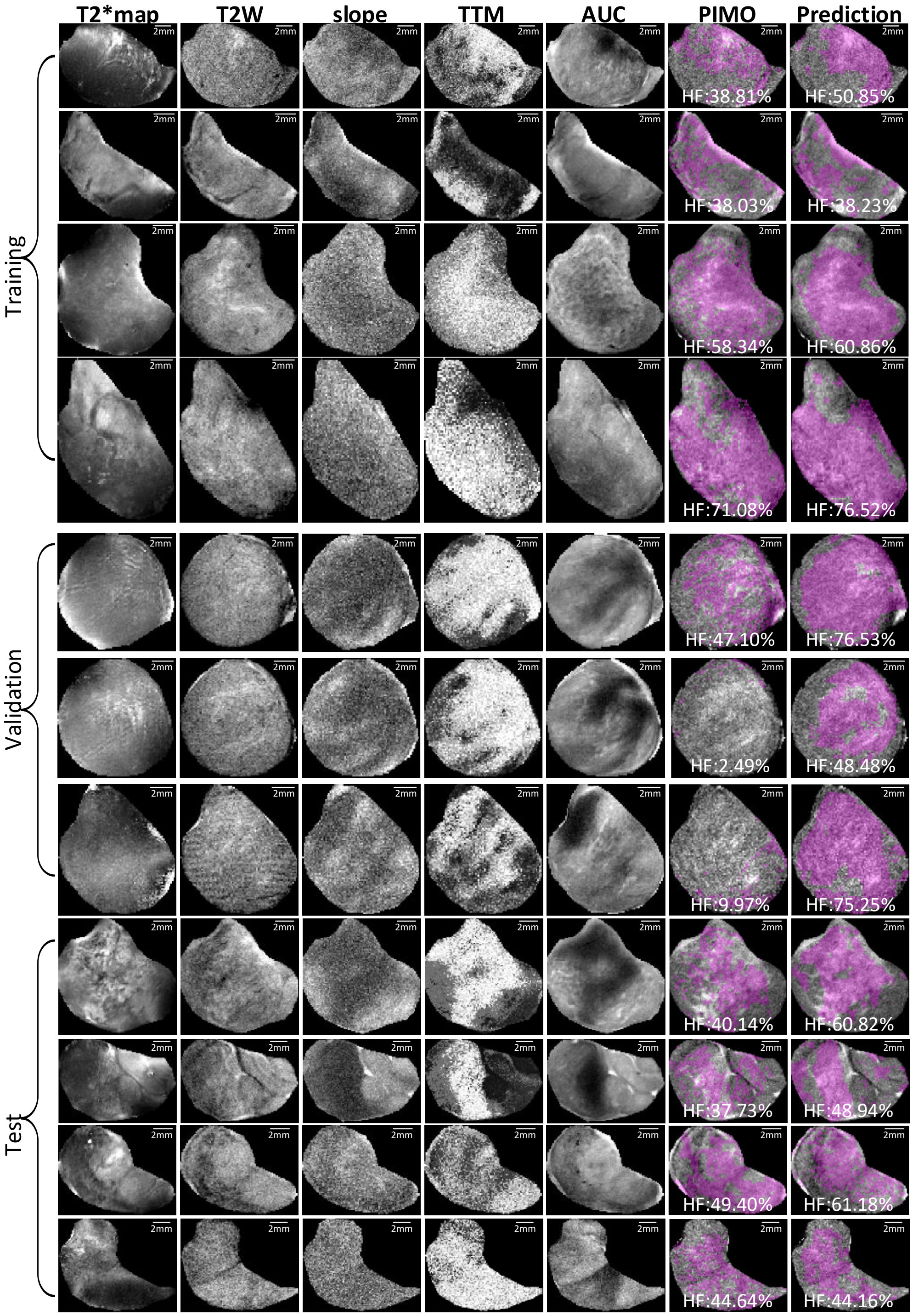


**Figure S6. Additional samples of training, validation and test datasets for patient-derived xenograft (PDX) rhabdomyosarcoma model.** Images shows multiparametric MRI maps ($T_2$* map, $T_2$-weighted image ($T_2$W) and slope, time to maximum (TTM) and area under the curve (AUC) from dynamic contrast enhanced (DCE) MRI), co-registered pimonidazole stained histology slice (PIMO), and predicted hypoxia fraction (prediction). Note. HF means hypoxia fraction.

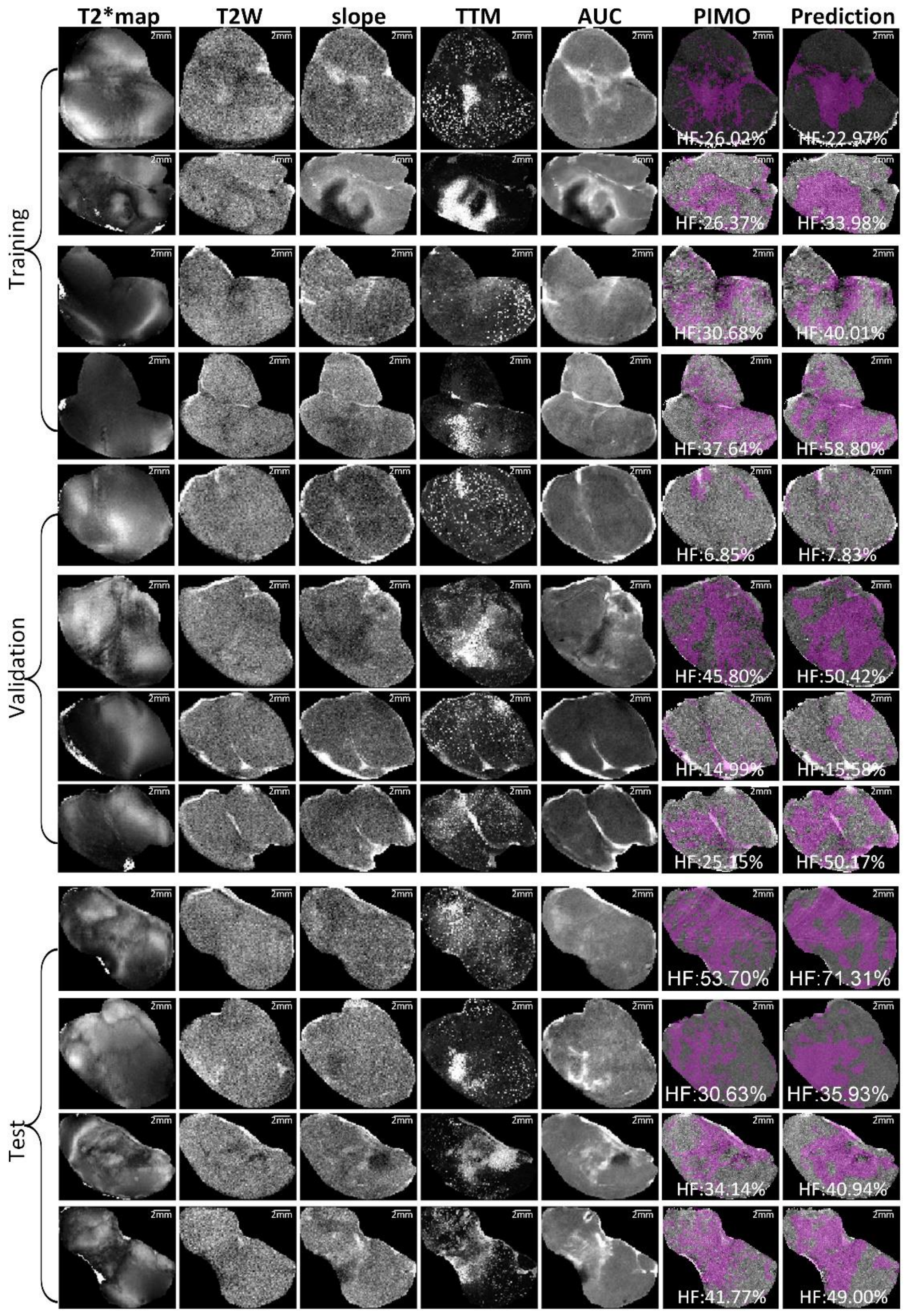


**Figure S7. Additional samples of training, validation and test datasets for radiation-induced fibrosarcoma (RIF-1) tumors.** Images shows multiparametric MRI maps ($T_2$* map, $T_2$-weighted image ($T_2$W) and slope, time to maximum (TTM) and area under the curve (AUC) from dynamic contrast enhanced (DCE) MRI), co-registered pimonidazole stained histology slice (PIMO), and predicted hypoxia fraction (prediction). Note. HF means hypoxia fraction.

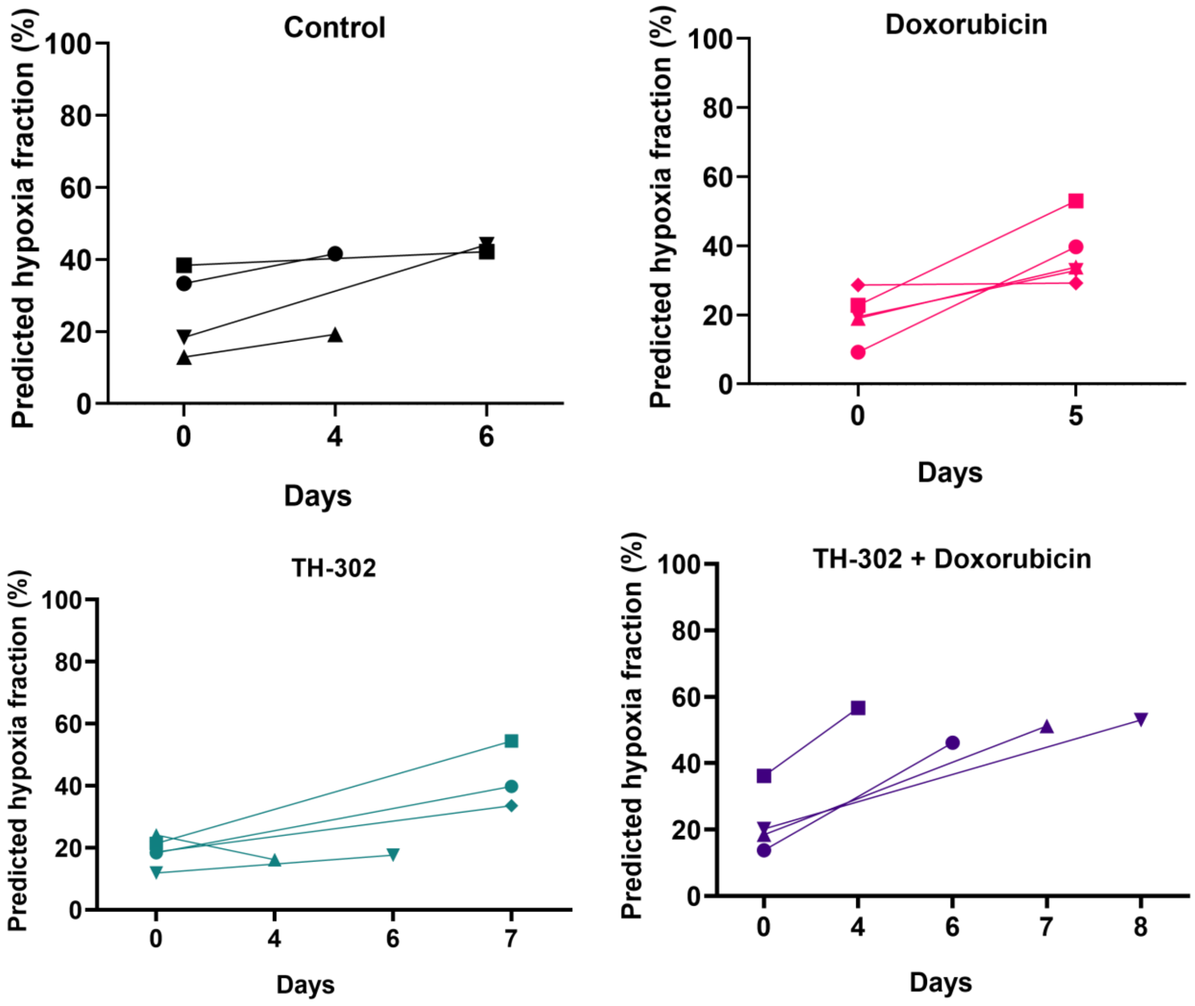


**Figure S8.** Pre- and post-therapy measurements of predicted hypoxia fraction in MRI for the RIF-1 tumor model.

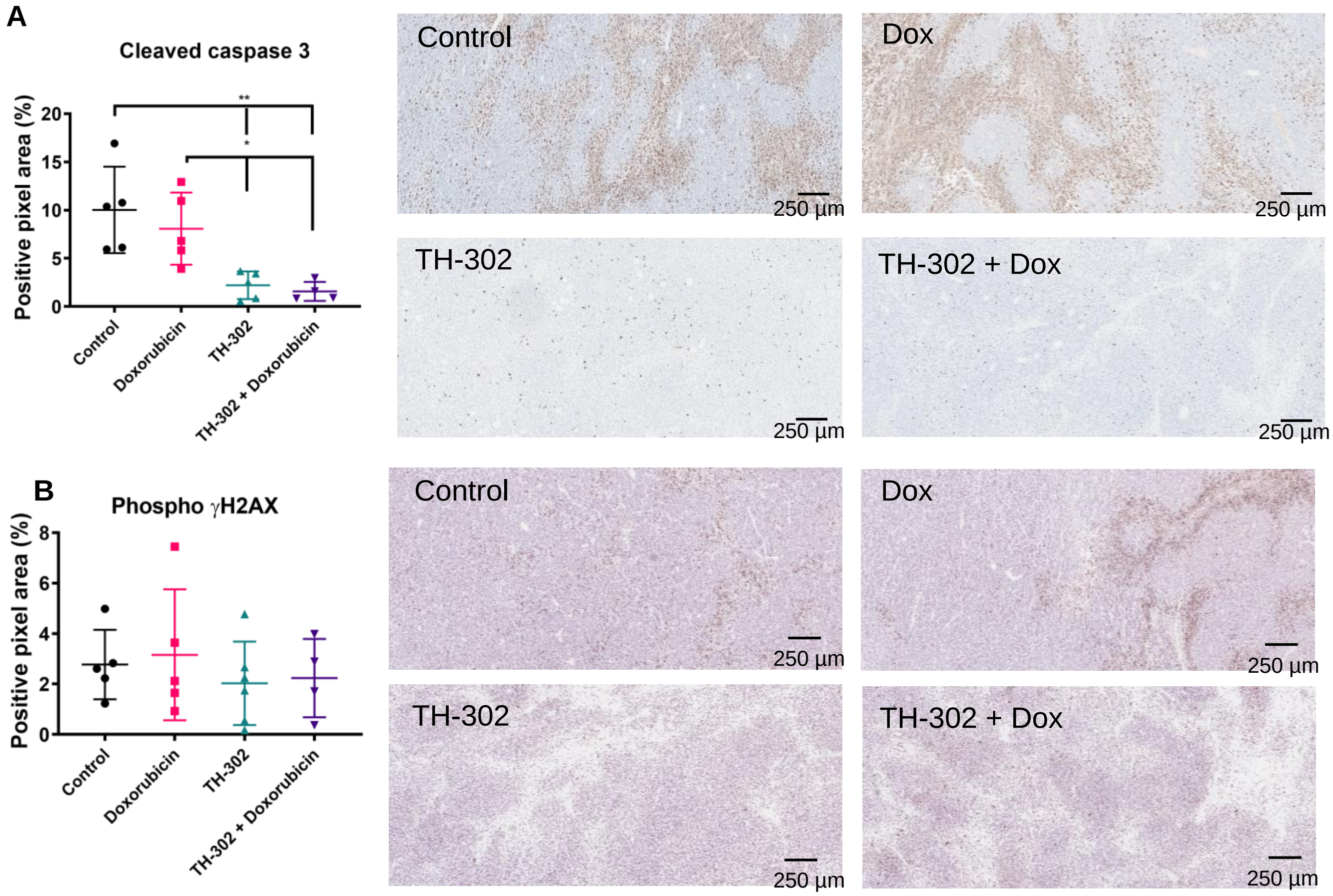


**Figure S9. Quantification and representative images for patient-derived xenograft (PDX) rhabdomyosarcoma tumors stained with DNA-damage marker (phospho γ-H2AX) and apoptosis marker Cleaved Caspase 3 (CC3). A.** CC3 comparisons (p>0.76 Doxorubicin (Dox) *vs* Control; **p=0.006 TH-302 *vs* Control; **p=0.005 TH-302 + Dox *vs* Control; *p=0.04 TH-302 *vs* Dox; *p=0.03 TH-302 + Dox *vs* Dox; p=0.99 TH-302 *vs* TH-302 + Dox). **B.** Phospho γ-H2AX comparisons (p>0.98 Dox *vs* Control; p=0.98 TH-302 *vs* Control; p=0.97 TH-302 + Dox *vs* Control; p=0.89 TH-302 *vs* Dox; p=0.88 TH-302 + Dox *vs* Dox; p=0.99 TH-302 *vs* TH-302 + Dox). Analysis of variance (ANOVA) followed by Bonferroni multiple comparisons test.  Values presented as mean± SD.
